# Awareness and Knowledge About Preventive Vaccinations Among Patients with Hematological Malignancies

**DOI:** 10.3390/vaccines13030284

**Published:** 2025-03-07

**Authors:** Marta Morawska, Marta Masternak, Norbert Grząśko, Ewa Lech-Marańda, Tomasz Wróbel, Sebastian Giebel, Krzysztof Tomasiewicz, Krzysztof Giannopoulos

**Affiliations:** 1 Department of Experimental Hematooncology, Medical University of Lublin, 20-093 Lublin, Poland; mmorawska79@gmail.com (M.M.); podgorniakmarta@gmail.com (M.M.); norbertgrzasko@gmail.com (N.G.); 2 Department of Hematology, Institute of Hematology and Transfusion Medicine, 02-776 Warsaw, Poland; ewamaranda@wp.pl; 3 Clinical Department of Hematology, Cell Therapies and Internal Diseases, Wroclaw Medical University, 50-367 Wroclaw, Poland; tomasz.wrobel@umw.edu.pl; 4 Department of Bone Marrow Transplantation and OncoHematology, Maria Sklodowska-Curie National Research Institute of Oncology, Gliwice Branch, 44-102 Gliwice, Poland; sebastian.giebel@io.gliwice.pl; 5 Department and Clinic of Infectious Diseases and Hepatology, USK-1, Medical University of Lublin, 20-090 Lublin, Poland; krzysztof.tomasiewicz@umlub.pl

**Keywords:** hematological malignancies, vaccination, multiple myeloma, COVID-19, vaccine hesitancy

## Abstract

Background: Patients with hematological malignancies, including multiple myeloma (MM) and chronic lymphocytic leukemia (CLL), are at an increased risk of severe infections due to both disease- and therapy-related immunosuppression. This cross-sectional study evaluated awareness of infection risks and vaccination uptake among 150 adults with various hematological malignancies from major Polish centers. Methods: All participants completed a 30-item questionnaire capturing demographic data, treatment history, infection frequency, and vaccination attitude. Statistical analyses utilized Chi-square and Fisher’s exact tests, with *p* < 0.05 considered statistically significant. Results: Respondents had a median age of 57 years (range, 30–79), and 65.3% were female. MM was the most common diagnosis (64.7%), followed by CLL (4.0%) and other hematological malignancies (31.3%). Nearly all participants (99.3%) acknowledged their increased susceptibility to infections. Frequent infections (≥2 in the past 6 months) were significantly associated with transfusion dependency (*p* = 0.0001) and a history of hematopoietic stem cell transplantation (HSCT, *p* = 0.009). Although 69.3% expressed willingness to be vaccinated, 23.3% declined COVID-19 vaccination due to insufficient cancer-specific safety data. Higher education and urban residence correlated with greater acceptance of vaccines (*p* < 0.05). Conclusions: Our findings underscore the critical need for targeted educational strategies and robust vaccination guidelines in this immunocompromised population. Enhanced patient education and timely implementation of tailored vaccination regimens could reduce infection-related morbidity and improve the tolerability of cancer treatments.

## 1. Introduction

Hematological malignancies, comprising myeloid and lymphoid neoplasms, result from disruptions in normal hematopoiesis and include leukemia, multiple myeloma (MM), non-Hodgkin lymphoma (NHL), and Hodgkin lymphoma (HL) [1,2]. Chronic lymphocytic leukemia (CLL), the most prevalent leukemia in Europe and North America, is characterized by an indolent but clinically heterogeneous course, with neoplastic, phenotypically mature B lymphocytes accumulating in peripheral blood, bone marrow, and lymphoid tissues [3,4]. Multiple myeloma, a plasma cell malignancy, is marked by the clonal proliferation of malignant plasma cells, resulting in significant immunosuppression and end-organ damage [5]. In both CLL and MM, infectious complications remain the predominant cause of morbidity and mortality, driven by profound immunodeficiency associated with both the disease pathophysiology and its treatment modalities.

Immunosuppression in hematological malignancies is multifactorial, resulting from hypogammaglobulinemia, dysregulated immune cell subpopulations, and impaired T-cell effector functions [6,7]. B-cell-targeting therapies, including anti-CD20, anti-CD38 monoclonal antibodies, and Bruton’s tyrosine kinase (BTK) inhibitors, further compromise humoral immunity, exacerbating susceptibility to infections [8,9,10]. Additionally, hematopoietic stem cell transplantation (HSCT) and other intensive therapeutic interventions can significantly amplify immunosuppression [11,12]. These factors collectively contribute to suboptimal immunologic memory against vaccine antigens, underscoring the need for carefully timed and individualized vaccination protocols [13,14,15,16].

The COVID-19 pandemic has highlighted these vulnerabilities. Evidence shows that patients with CLL or MM are at substantial risk of severe disease and mortality from SARS-CoV-2, regardless of disease stage or treatment type [17,18,19]. Notably, anti–B-cell therapies have been shown to significantly attenuate serologic responses to vaccination, though cellular immunity may remain partially preserved [10,20]. These findings emphasize the critical need to balance risks and benefits when implementing preventive vaccination strategies against SARS-CoV-2, influenza, and other common pathogens. For high-risk subgroups, additional infection-prevention strategies, such as pharmacological prophylaxis or passive immunization, may also be necessary [20,21].

Despite the clinical importance of vaccination, patient awareness and acceptance of these strategies can vary. The increased risk of infections and frequent hospitalizations may drive some patients toward timely vaccination while fostering apprehension in others due to concerns about potential adverse effects. Clear, patient-centered education by healthcare providers is essential to improving adherence to vaccination recommendations. Effective communication can enhance patient understanding, mitigate vaccine hesitancy, and ultimately improve health outcomes by enabling better tolerance and efficacy of anticancer therapies.

In the era of digital technologies, when patients often encounter an overwhelming volume of information, ensuring access to accurate, disease-specific guidance is both challenging and essential. Strengthening patient knowledge and fostering engagement are key to promoting shared decision-making, safer treatment, and improved quality of life for individuals with hematological malignancies. Reliable studies are needed to assess patients’ awareness of the infectious threat and their attitudes toward preventive measures. The obtained data are a valuable clue to the unmet needs of hematological patients in terms of education and prevention.

### Objective

This study assessed the issues of infectious complications and awareness of preventive strategies and attitudes toward them among hematology patients. The objectives of the study were to assess awareness of the infectious threat, the frequency and course of infections, vaccination coverage, and attitudes toward vaccinations of the study population.

## 2. Materials and Methods

### 2.1. Study Design and Participants

This cross-sectional study enrolled 150 patients treated for hematological cancers in various cities in Poland. Survey data were collected between December 2023 and July 2024. The survey was distributed by members of the patient organization in the form of a paper questionnaire. Ethical approval was obtained from the local Bioethical Committee of the Medical University of Lublin (approval number KE-0254/345/2019), and all participants provided written informed consent prior to inclusion.

### 2.2. Questionnaire

Data were collected using a structured, 30-item questionnaire, divided into four sections:

Demographic Data: Age, sex, education, place of residence, diagnosis, performance status, and prior treatment history.

Occurrence of Infections: Frequency and severity of infections experienced.

Prevention of Infections: Strategies and behaviors adopted to mitigate infection risk.

Vaccinations: Immunization history and attitudes toward vaccination.

All survey questions were closed-ended, and responses were analyzed as categorical variables. The studied parameters included variables dependent on the respondents, such as attitude towards vaccination, willingness to expand knowledge, and independent variables, such as the occurrence of frequent infections, previous treatment, etc.

### 2.3. Statistical Analysis

Frequencies and percentages were used to describe the categorical variables. Statistical analyses were conducted using GraphPad Prism (version 10.2.3). The Chi-square test for trends was applied to assess variations in willingness to accept COVID-19 vaccination. Correlation analyses between selected variables (e.g., hematological malignancy type, treatment status, and transfusion dependency) and vaccination attitudes were performed using the Chi-square test of independence or Fisher’s exact test, as appropriate. In specific subgroup analyses, one-sample t-tests and Wilcoxon tests were used. Statistical significance was defined as a two-sided *p* < 0.05.

## 3. Results

### 3.1. Background Characteristics

The study cohort included 150 patients, with a median age of 57 years (range, 30–79). Female patients comprised 65.3% of the sample. MM was the most common diagnosis, accounting for 64.7% of cases, followed by CLL in 4.0% and other hematological malignancies in 31.3%.

Performance status was assessed using the Eastern Cooperative Oncology Group (ECOG) scale, with no participants scoring 3 or 4. Of the total cohort, 54.7% had a score of 0, indicating normal performance and full independence, 32.7% had a score of 1, reflecting symptomatic but ambulatory status capable of light work, and 12.6% had a score of 2, denoting the ability to perform self-care but an inability to work, with less than half the day spent in bed.

**Figure 1:**
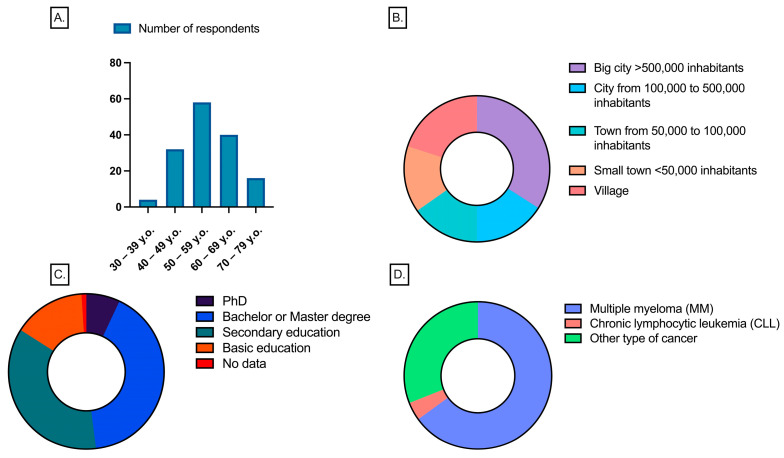
General description of study participants: (**A**) age distribution; (**B**) dwelling place; (**C**) educational level; (**D**) diagnosis. The graph was created in GraphPad Prism version 10.2.3.

Regarding treatment history, 82.6% of patients had undergone therapy, including 30.7% who received pharmacological treatment only and 52.0% who underwent HSCT. The remaining 17.3% had not received any hematological treatment. Blood product transfusions were reported by 45.3%, while 52.7% had never received a transfusion, and three participants declined to answer. The overall composition of the study group is illustrated in Figure 1.

### 3.2. Frequency and Course of Infection

Nearly all patients (99.3%) acknowledged that a hematological diagnosis increases their susceptibility to infections. Among the total cohort, 64.6% reported frequent upper respiratory tract infections, with 58.7% indicating they experienced infections at least twice in the past six months. Patients diagnosed with MM were significantly more likely to report frequent infections (79.4%) compared to those with other hematological malignancies (37.7%).

Patients requiring regular blood product transfusions reported frequent infections more often than those who did not (81.4% vs. 51.9%, *p* = 0.0001). This correlation was significant in both MM patients (*p* = 0.002, Fisher’s exact test) and those with other hematological malignancies (*p* < 0.0001, Fisher’s exact test). Similarly, patients who had undergone HSCT reported higher infection rates than those treated without HSCT or untreated patients (78.2% vs. 45.7% vs. 57.7%, respectively; *p* = 0.009).

No significant association was observed between performance status (ECOG 0–2) and the frequency of infections (*p* = 0.8011, Fisher’s exact test). Additionally, the frequency of infections did not correlate with participants’ vaccination attitude (*p* = 0.7552, Fisher’s exact test).

Regarding infection severity, 31.4% of respondents reported prolonged infections that sometimes required antibiotics, while 17.3% indicated prolonged infections always requiring antibiotics. In contrast, 27.3% described their infections as “usually mild”, and 24.0% stated they were “always mild”. Overall, 48.7% of participants experienced prolonged infections, while 51.3% reported milder courses. No significant differences in infection severity were identified based on diagnosis type, treatment approach, or transfusion status.

### 3.3. Vaccinations

When asked about general vaccination (for any available vaccine), 69.3% of participants expressed willingness to be vaccinated. Willingness was significantly higher among individuals residing in larger cities (78.7%) compared to those in smaller towns (66.7%) or villages (50.0%; *p* = 0.0158, Fisher’s exact test). A similar trend was observed for educational level, with 82.2% of participants with higher education expressing willingness, compared to 59.3% with secondary education and 50.0% with primary education (*p* = 0.0047, Fisher’s exact test).

However, patients who had undergone HSCT were less likely to accept vaccination compared to those without HSCT or untreated patients (61.5% vs. 80.4% vs. 70.3%; *p* = 0.0154, Fisher’s exact test).

### 3.4. COVID-19 Vaccination

Among the cohort, 76.7% chose to receive a COVID-19 vaccine. Of these, 62.0% sought vaccination at the earliest opportunity, 11.3% were initially hesitant but decided to vaccinate due to cancer-related risks, and 3.0% followed their physician’s recommendation.

No significant correlation was found between disease type and the decision to vaccinate against COVID-19. Similarly, no significant association was observed between overall educational level and COVID-19 vaccination decisions (*p* = 0.1366, Chi-square test) in the entire cohort or among MM patients. However, within the subgroup of other hematological malignancies, patients with higher education were more likely to accept vaccination than those with primary or secondary education (*p* = 0.0408, Fisher’s exact test). Place of residence did not significantly influence COVID-19 vaccination decisions in any subgroup.

Patients dependent on transfusions were more likely to opt for COVID-19 vaccination than those who were not (*p* = 0.0160, Chi-square test). Among the 23.3% who declined COVID-19 vaccination, the primary reasons included the lack of knowledge about vaccine effects in cancer patients (73.0%), concerns about exacerbating their disease (17.6%), and apprehension regarding vaccine tolerance (8.8%).

These findings underscore the complex interplay between clinical characteristics, patient perceptions, and vaccination decisions in this vulnerable population.

**Figure 2:**
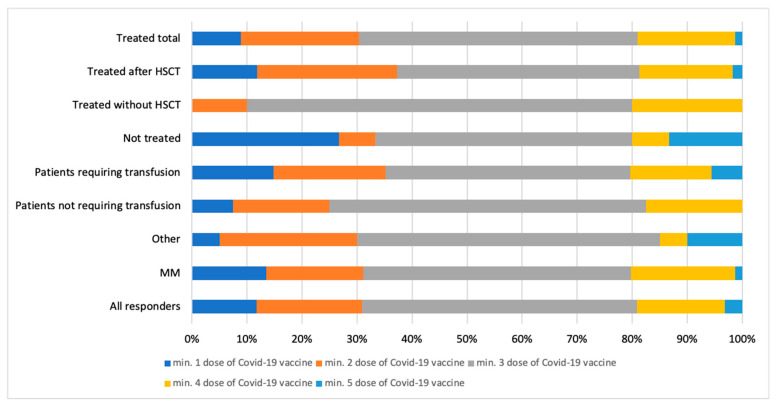
COVID-19 vaccine distribution in the analyzed subgroups.

The distribution of COVID-19 vaccine doses administered is presented in Figure 2.

### 3.5. Sources of Information About COVID-19 Vaccination

Healthcare workers were the primary source of information about COVID-19 vaccination for most respondents, with 64.7% receiving guidance from their oncology specialist and 16.7% from their primary care physician. Other information sources included traditional media (press, radio, television) in 37.3% of cases, social media in 10.0%, and family or friends in 31.3%.

Regarding booster doses of the COVID-19 vaccine, only 20.7% of respondents believed that additional doses were necessary, while 56.7% denied the need for further vaccination, and 22.6% were undecided. Among vaccinated respondents who cited television or other mass media as their primary source of information (11.3%), the majority expressed uncertainty about the necessity of booster doses.

### 3.6. Other Vaccinations

Influenza vaccination within the previous four years was reported by 54.0% of respondents, with 37.3% having received the vaccine during the most recent influenza season. Neither educational level nor place of residence significantly influenced influenza vaccination uptake.

Prophylactic vaccinations against other pathogens, including hepatitis A, hepatitis B, varicella-zoster virus, and pneumococcus, were reported by 44.7% of participants. However, 32.7% indicated that they were aware of these vaccines but had not received a formal recommendation to undergo immunization, while 22.0% refused such vaccinations due to concerns about a potential worsening of their disease. Participants who opted for COVID-19 vaccination were significantly more likely to have received other prophylactic vaccines (*p* < 0.0001, Chi-square test).

### 3.7. Education and Awareness

The majority of respondents (84.0%) expressed willingness to attend in-person educational events about their disease if organized locally. Additionally, 65.3% indicated an interest in participating in similar events specifically focused on infection prevention and vaccinations. The willingness to expand knowledge about vaccinations was more common among vaccinated (68.7%) than among unvaccinated patients (54.3%), but the result was not statistically significant (*p* = 0.1552).

## 4. Discussion

This study highlights the high burden of infections among Polish patients with hematological malignancies. The results show a high awareness of the increased risk of infection and the implementation of adequate preventive measures in a significant percentage of this particular group of patients. Most survey respondents were aware of their increased exposure to bacterial and viral diseases and observed frequent infections. Most of the survey respondents observed frequent infections (≥2 in the past six months) that were significantly more common among patients with a history of HSCT compared to those without HSCT and untreated patients. These findings are consistent with the report by Robin et al. [22], who reported high respiratory viral infection rates in stem cell transplant recipients, thereby underscoring the need for vigilant monitoring and prevention strategies in this population. These findings emphasize the profound impact of clinical factors, such as HSCT and transfusion dependency, on infection susceptibility, underscoring the need for proactive infection prevention strategies.

Clinical factors—particularly transfusion dependency and prior HSCT—were also significantly associated with vaccination decisions in this cohort. While a higher percentage of individuals with higher education and those residing in urban areas demonstrated greater willingness to vaccinate, actual COVID-19 vaccination rates were not significantly different across these groups. This suggests that patients with lower education levels may be more influenced by recommendations from healthcare professionals. This is underscored by the fact that 64.7% of respondents identified their oncologists or family physicians as their primary source of vaccination information. Similarly, findings from the Leukemia & Lymphoma Society survey support this conclusion, showing that rural and suburban residents tend to exhibit higher vaccine hesitancy but may still be influenced positively by trusted healthcare providers [23]. These findings emphasize the critical role of healthcare professionals in addressing vaccine hesitancy and ensuring equitable vaccine uptake, particularly among populations with limited access to educational resources.

The impact of healthcare professionals on vaccination uptake aligns with findings from Tsai et al., who reported that lower education levels were associated with higher vaccine hesitancy, but trusted communication from healthcare providers played a significant role in mitigating hesitancy in patients with chronic illnesses [24]. This reinforces the importance of targeted education campaigns that leverage the role of trusted medical professionals to improve vaccine acceptance, especially among patients with limited educational backgrounds.

Vaccination rates in this cohort exceeded those observed in the general Polish population for both COVID-19 and other preventive vaccinations. While only 5.5% of the general population received the influenza vaccine during the previous infectious season, 37.3% of this cohort reported vaccination [25]. Similarly, 76.7% of patients in this study received at least one dose of the COVID-19 vaccine, compared to 64.1% in the general population [25]. These results likely reflect heightened awareness of infection risks and proactive engagement with healthcare professionals, who played a central role in encouraging preventive measures.

These results align with prior literature emphasizing the importance of vaccination in reducing infection risk, particularly in long-term hematologic oncology survivorship [2]. Patients with CLL and MM are particularly vulnerable due to multifaceted immunosuppression resulting from the underlying disease, the tumor microenvironment, and therapies such as BTK inhibitors or monoclonal antibodies [1,6,7,8,10,13]. Consequently, these patients face elevated risks of severe infections and hospitalizations [5,17,18,19]. Prophylactic vaccination offers a critical tool to mitigate these risks but often requires tailored schedules and repeated doses to address suboptimal or waning immune responses [9,10,11,12].

### 4.1. Efficacy and Durability of COVID-19 Vaccine Responses

Evidence demonstrates that vaccine-induced immunity varies significantly by disease subtype and treatment status. A recent study by Nutor et al. [26] examined the immunogenicity of COVID-19 vaccination post-CAR T therapy in patients with Non-Hodgkin Lymphoma (NHL) and Multiple Myeloma, finding that although some immune response was detectable, the overall seroconversion rates were notably lower compared to immunocompetent individuals. The timing of vaccination relative to CAR-T-cell infusion and the degree of B-cell reconstitution were highlighted as critical factors influencing vaccine efficacy necessitating earlier booster doses. Similarly, Zaleska et al. [27] reported that while MM patients generally achieve stable humoral responses to mRNA vaccines, CLL patients—especially those undergoing active therapy—exhibit weaker short-term serologic responses and similar long-term waning.

Real-world data corroborate these findings. Morawska [28] highlighted significant vaccine failure rates in CLL patients, largely attributable to disease- and treatment-induced immune dysfunction. This underscores the need for personalized vaccination strategies, including considerations for alternative schedules, booster doses, and passive immunization approaches. The European Research Initiative on CLL (ERIC) further emphasizes the importance of booster doses, highlighting that T-cell-mediated immunity remains clinically relevant even when serologic responses are suboptimal [9,17,18,19].

Additionally, ERIC’s study on the evolving landscape of post-COVID conditions in patients with CLL underscores the long-term impact of SARS-CoV-2 on this immunocompromised group [29]. Their findings stress the necessity of integrating post-infection care and tailored vaccination strategies to improve outcomes and mitigate severe post-COVID complications.

In our study, 23.3% of patients declined COVID-19 vaccination, citing concerns about side effects (17.6%), limited oncology-specific safety data (73.0%), and vaccine tolerance (8.8%). Similarly, a survey by the Leukemia & Lymphoma Society found 17% vaccine hesitancy, primarily driven by concerns about side effects (54%) and doubts about proper vaccine testing (54%) [23]. These findings highlight the urgent need for tailored educational interventions that directly address these specific concerns and provide reliable, evidence-based information on vaccine safety and efficacy.

These insights emphasize the necessity of strengthening patient–provider communication, particularly for vulnerable individuals with limited access to accurate medical information. Leveraging the influence of healthcare professionals through targeted educational campaigns can play a pivotal role in reducing vaccine hesitancy and improving acceptance rates in high-risk populations such as patients with hematological malignancies.

### 4.2. Beyond COVID-19: Prophylactic Vaccinations

Seasonal influenza and other prophylactic vaccines were widely adopted by respondents, with uptake rates surpassing those observed in the general Polish population. Studies by Ljungman et al. [30] and Ludwig et al. [31] have similarly demonstrated that immunosuppressed individuals benefit from tailored immunization schedules to mitigate severe infection risks. Moreover, healthcare professionals played a crucial role in vaccination uptake in this cohort, with most patients citing their oncologists or family physicians as the primary sources of information.

Further emphasizing the need for proactive vaccination strategies, findings from the Polish Adult Leukemia Group (PALG) identified a significant risk of pneumonia in CLL patients receiving Venetoclax-based regimens. Vaccines targeting pneumococcus and influenza are particularly important for mitigating infection risks in these vulnerable populations [32].

The current guidelines, including those from ASCO, advocate for vaccination against pathogens such as influenza, RSV, pneumococcus, hepatitis B, varicella-zoster, and HPV in adults with cancer [20,33]. Prophylactic immunizations should ideally be administered before initiating cancer therapy or during periods of minimal treatment-induced immunosuppression [14,15,16,21]. Emerging therapies, such as CAR T-cell treatments, may further complicate vaccine responses, underscoring the need for individualized vaccination schedules and potentially higher doses [26].

### 4.3. Clinical Implications and Future Directions

Our findings demonstrate that hematological patients, particularly those with higher education or living in urban areas, show relatively strong adherence to vaccination schedules. Nonetheless, many participants indicated a need for clearer and more frequent guidance from healthcare professionals regarding booster doses and emerging vaccines. Educational initiatives are a compelling opportunity to enhance patient engagement, with 84% of respondents expressing willingness to attend local events on their disease and 65% open to sessions focused on infection prevention and vaccination.

Future research should focus on optimizing vaccination regimens tailored to specific hematological malignancies, accounting for disease stage, therapy type, and individual immunologic profiles. Longitudinal studies measuring antibody titers, T-cell activity, and real-world outcomes, such as infection rates and hospitalizations, are needed to refine evidence-based vaccination strategies [32,34]. Such efforts could yield patient-centered guidelines that balance oncologic treatment urgency with the necessity of preemptive or concurrent vaccination [20,33,35].

### 4.4. Limitations

There are some potential limitations to the study that need to be considered. One of them is the use of convenience sampling, which might be a drawback in inferring the results on the whole population. In addition, the study is based on a self-administered questionnaire, which could potentially affect the reliability of the responses. Nevertheless, the study included a large group of patients with hematological diagnoses, providing a valuable perspective on one of the most common complications of oncological treatment.

## 5. Conclusions

This study demonstrates that most patients with hematological malignancies recognize their heightened infection risk and exhibit relatively high vaccine uptake, particularly for COVID-19. However, a significant minority of responders remain unvaccinated, often due to concerns about vaccine safety and insufficient knowledge about the impact of vaccination on their disease. Clinical factors, such as transfusion dependency and prior HSCT, strongly correlate with vaccination decisions. Although educational level and place of residence were not statistically significant overall, trends suggest that individuals with higher education and urban residency have greater awareness and acceptance of vaccination.

These findings underscore the critical need for tailored patient education on the benefits of vaccination and the importance of booster doses. Real-world data further emphasize the challenges posed by disease- and treatment-induced immune dysfunction, particularly in patients with chronic lymphocytic leukemia, highlighting the importance of personalized vaccination schedules and additional prophylactic measures. By integrating evidence-based vaccination guidelines into standard hematology-oncology care, healthcare providers can help mitigate infection-related morbidity and mortality.

Future research should prioritize optimizing vaccination strategies tailored to specific hematological malignancies, accounting for disease stage, therapy type, and immunologic profiles. Longitudinal studies focusing on immunologic outcomes, such as antibody titers and T-cell activity, and real-world measures, such as infection rates and hospitalizations, will be crucial. These efforts could yield patient-centered guidelines that balance the urgency of oncologic treatment with the necessity of preemptive or concurrent vaccination, ultimately improving outcomes for this vulnerable population.

## 6. Key Points

Increased Infection Risk Awareness: 99.3% of patients acknowledged their heightened susceptibility to infections due to disease- and therapy-related immunosuppression.

High Infection Burden: Frequent infections were significantly more common in patients with multiple myeloma, transfusion dependency, or prior HSCT, highlighting the importance of tailored infection prevention strategies.

Vaccination Uptake: 76.7% of patients received COVID-19 vaccines, and 69% were vaccinated against at least one additional pathogen, exceeding rates in the general population.

Barriers to Vaccination: Among the 23.3% unvaccinated, primary concerns included safety, insufficient oncology-specific data, and potential side effects.

Role of Healthcare Providers: Trusted guidance from oncologists and family physicians emerged as a critical factor in overcoming vaccine hesitancy, particularly among patients with lower educational backgrounds.

Recommendations: Proactive patient education, personalized vaccination schedules, and research into immunogenicity in hematological malignancies are essential to enhancing vaccine coverage and reducing infection-related morbidity.

## Data Availability

The anonymous data presented in this survey are available upon request from the corresponding author.
